# Economic assessment of incorporating the hexavalent vaccine as part of the National Immunization Program of Peru

**DOI:** 10.1186/s12913-022-08006-1

**Published:** 2022-05-16

**Authors:** Janice Seinfeld, María Laura Rosales, Alfredo Sobrevilla, Juan Guillermo López Yescas

**Affiliations:** 1 Videnza Consultores, Calle Alberto Alexander 2695, Lince, Lima, Perú; 2 Sanofi, Mexico City, Mexico

**Keywords:** Budget impact analysis, Cost-minimization analysis, Combination vaccines, Acellular hexavalent vaccine, Oral poliovirus vaccine, Peru, Public health policy, Vaccination, Expanded Program on Immunization, Primary vaccination scheme

## Abstract

**Background:**

This study aimed to estimate the economic impact of replacing the current Peruvian primary immunization scheme for infants under 1 year old with an alternative scheme with similar efficacy, based on a hexavalent vaccine.

**Methods:**

A cost-minimization analysis compared the costs associated with vaccine administration, adverse reactions medical treatment, logistical activities, and indirect social costs associated with time spent by parents in both schemes. A budgetary impact analysis assessed the financial impact of the alternative scheme on healthcare budget.

**Results:**

Incorporating the hexavalent vaccine would result in a 15.5% net increase in healthcare budget expenditure ($48,281,706 vs $55,744,653). Vaccination costs would increase by 54.1%, whereas logistical and adverse reaction costs would be reduced by 59.8% and 33.1%, respectively. When including indirect social costs in the analysis, the budgetary impact was reduced to 8.7%. Furthermore, the alternative scheme would enable the liberation of 17.5% of national vaccines storage capacity.

**Conclusions:**

Despite of the significant reduction of logistical and adverse reaction costs, including the hexavalent vaccine into the National Immunization Program of Peru in place of the current vaccination scheme for infants under 1 year of age would increase the public financial budget of the government as it would represent larger vaccine acquisition costs. Incorporating the indirect costs would reduce the budgetary impact demonstrating the social value of the alternative scheme. This merits consideration by government bodies, and future studies investigating such benefits would be informative.

**Supplementary Information:**

The online version contains supplementary material available at 10.1186/s12913-022-08006-1.

## Background

The National Immunization Program of the Peruvian Ministry of Health (Ministerio de Salud de Perú [MINSA]) was established in 1976 to protect the general population against vaccine-preventable diseases [1] and was most recently updated in 2018 [2]. As part of this strategic healthcare intervention, all infants under the age of 1 year should be primary vaccinated against diphtheria, tetanus, hepatitis B, *Haemophilus influenzae* type B (Hib), and poliomyelitis by the administration of three doses of a pentavalent vaccine (DTwP-HB-Hib) plus two doses of the inactivated polio vaccine (IPV) and one dose of the oral polio vaccine (OPV) [2]. These six diseases are associated with significant morbidity and mortality, particularly in infants, and maintaining high rates of immunization coverage is crucial to avoid their spread [3–8].

A hexavalent vaccine (DTaP-HB-Hib-IPV) that prevents all six diseases has been prequalified by the WHO [9]. Various clinical trials performed in Latin America have reported its high level of immunogenicity (> 95%) [10, 11] and, it is already part of national vaccination programs across most European countries, as well as Canada, Panama, and Chile [12–15]. In Peru, its use has been approved, although it is only available in the private market [16].

The composition of the hexavalent vaccine [17] provides several benefits, most importantly the fact that it eliminates the risk of contracting vaccine-derived poliovirus [18]. The OPV utilizes a live-attenuated poliovirus and presents a high risk of fecal–oral transmission, increasing the potential for cases of circulating vaccine-derived poliovirus (cVDPV) [19]. Therefore, the current position of the WHO is that successful eradication of polio depends upon complete withdrawal of OPV vaccines [20]. Given its IPV component, the hexavalent vaccine represents an alternative to stop the occurrence of vaccine-associated paralytic poliomyelitis (VAPP) and achieving this aim [18].

In addition, being a combination vaccine, the hexavalent vaccine could reduce the burden of multiple injections and provide better acceptance from parents [21], in turn improving compliance and timeliness of vaccination [22]. Nevertheless, a more integrated scheme also entails some limitations such as greater sensibility to supply interruptions [23] and higher acquisition prices [24].

Considering Peruvian healthcare budget constraints and the importance of strengthening financial sustainability for immunization programs, the evaluation of the economic impact of incorporating this new scheme in Peru is of high interest for the payer. Because the two schemes have been shown to have similar efficacy, the following comprehensive economic assessment consists of a cost-minimization analysis (CMA) comparing the costs associated with the current Peruvian primary immunization scheme with those of the hexavalent-based scheme, from a social perspective. Furthermore, a budgetary impact analysis (BIA) was carried out to evaluate the financial impact of replacing the current primary immunization scheme with the hexavalent vaccine-based scheme on healthcare budget, in the context of the Peruvian Immunization Program.

## Methods

### Current vaccination scheme (existing scenario)

Characteristics of the pentavalent, IPV, and OPV vaccines are detailed in Supplementary Table 1 [2].

For the purpose of the economic assessment and based on interviews with management and operational experts (MINSA budget specialist, a pediatrician of the National Institute of Child Health, and a nurse responsible of the immunization program at a vaccination center), 95% of vaccines were assumed to be administered within the Peruvian Health Service (establecimientos de salud [EESS]), with the remaining 5% administered outside of the EESS during activities such as vaccination campaigns and home visits. Vaccination uptake rates reported in the Demographic and Family Health Inquest-Endes 2017 (Encuesta Demográfica y de Salud Familiar) were also considered in the economic model: 92.4%, 85.7%, and 77.2% for the first, second, and third pentavalent doses; 94.8% and 87.7% for the first and second IPV doses, and 78.6% for the first OPV dose [25]. Given deviation from guidelines recommending administration of both pentavalent and polio vaccines during the same medical visit, based on Endes 2017, the analysis also estimated ~ 5% of infants receiving pentavalent and polio vaccines at separate visits [25].

### Alternative vaccination scheme (novel intervention)

Characteristics of the hexavalent vaccine are detailed in Supplementary Table 1 [17]. As with the existing scheme, based on expert interviews, 95% of the vaccines were assumed to be administered within the EESS, with the remaining 5% administered outside of the EESS. To ensure comparability of the existing and novel scenarios, vaccination uptake rates reported for the current scheme [25] were also applied to the hexavalent vaccine.

### Target population

The National Immunization Program of Peru aims to vaccinate all infants < 1 year of age with primary doses of the pentavalent and polio vaccines. Therefore, this group represents the target population for the present cost estimations. According to the 2007 and 2017 national censuses and considering the proportion of the population not represented in these surveys, the estimated target population was ~ 500,000 individuals (51% male; 49% female) [26].

### Time horizon

All vaccines assessed are administered within the first year of life; [2] therefore, the time horizon for the present analysis was 1 year. This will capture the majority of local and systemic adverse reactions, which usually appear within hours to days of vaccine administration [2, 27–29].

One year is sufficient to identify cases of OPV-derived polio, which has an incubation period of 4‒40 days [30]. While individuals with polio may require > 16 years of rehabilitation, [31] the present analysis captures the costs associated with initial evaluation and treatment during the acute phase (first 6 months) [32].

### Cost calculations

The CMA and BIA presented in this paper follow the standard methodology for economic assessments, which are described below.

#### CMA – Costs per child vaccinated

CMA is a commonly used method for projecting the least costly therapeutic when, as in this case, alternative interventions have been proven to be equivalent in terms of all relevant outcomes [33]. The evaluation was made from a social perspective, [34] taking into account not only healthcare costs to administer the vaccines and to treat any resulting adverse reaction, but also considering related storage costs, and the indirect social cost valuing the time of parents to fully immunize their infants [35]. Average costs to fully protect an infant were calculated considering the following four sub-costs: logistical costs (including national planning, vaccine acquisition, storage, and distribution), vaccination costs (including personnel, medical equipment, devices and supplies, infrastructure, medical support services, and administrative expenses), adverse reaction costs (including the cost to identify, confirm, and treat local and systemic reactions and OPV-derived polio), and social costs (the value of caretaker’s time for medical services and transportation). The average considered all the scenarios by which the child could be fully immunized, and that the same child could experience all types of adverse reactions, because the occurrence of one reaction is independent from the others. Costs are reported in US dollars (USD, $), with $1 equal to S/ 3.374 Peruvian Soles (average accounting exchange rate reported on December 31, 2018 by the Peruvian Superintendence of Banks, Insurance and Private Pension Fund Administrators [Superintendencia de Banca, Seguros y AFP]) [36]. Additional cost calculation methods are included in the Supplementary Appendix.

#### BIA – Total cost estimation

BIAs are complementary analyses that provide an estimation of the financial impact of adopting a novel intervention compared with maintaining the existing scenario [37, 38]. The present BIA was carried out in line with recommendations from the International Society for Pharmacoeconomics and Outcomes Research (ISPOR), [39] adopting MINSA’s perspective. This entity is the governing body of the Peruvian National Immunization Program, responsible for ensuring the effective use of the program budget allocated by the Ministry of Economy and Finances. The BIA assessed the financial impact of replacing the current National Immunization Program primary vaccination scheme (pentavalent vaccine plus IPV and OPV; existing scenario) with the alternative vaccination scheme (the hexavalent vaccine only; novel intervention).

To extend the costs borne by the payer to implement the national immunization strategy, unitary costs previously calculated – with the exception of social costs – were considered and multiplied by the number of infants in the target population. For each vaccination scheme, the annual cost was calculated as a sum of the following three sub-costs: logistical, vaccination, and treatment of adverse reaction costs. For logistical cost estimations, vaccination of the entire target population was assumed. For all other sub-costs, only the applicable proportions of the target population were considered in calculations, according to the immunization or clinical variants identified. Finally, the annual costs of each vaccination scheme were compared to establish the estimated budgetary impact of the alternative scheme.

Methods for cost calculations and sensitivity analysis can be found in online supplementary methods.

### Ethics

We did not submit this study proposal for ethics committee review as no human subjects were involved. Such is the requirement stated by Peruvian regulations, according to the Technical Document: “Ethical Considerations for Health Research with Human Beings”, approved by Ministerial Resolution No. 233–2020-MINSA. We used de-identified secondary information, obtained from open access online platforms or via request for information to the corresponding public entities. Thus, all data is anonymous, ensuring our compliance with the “Personal Data Protection Law” and its complementary regulations (Law N° 29,733), approved by Supreme Decree No. 003–2013-JUS.

## Results

### Cost-minimization analysis

The estimated total cost of fully protecting one child using the hexavalent vaccine would be 8.7% higher than the cost required under the current scheme, increasing from $116.27 to $126.42. When considering this total by cost category, vaccination costs were the main driver for this increase, with the other components representing cost savings (Table 1).

**Table 1:** Estimated average cost by child vaccinated (USD, $), according to cost category

Cost category	Current scheme	Alternative scheme	% Difference
**Logistical costs**	**5.80**	**2.33**	**–59.8%**
**Vaccination costs**	**60.86**	**98.66**	** + 62.1%**
**Cost of treatment for adverse reactions**	**11,293.89**	**28.22**	**–99.8%**
Cost of treatment of common adverse reactions	37.75	28.22	–25.3%
Cost of treatment of OPV-derived polio	11,256.14	–	–100.0%
**Social cost of lost time**	**1,289.65**	**17.50**	**–98.6%**
Cost of time lost due to vaccination	9.31	8.22	–11.7%
Cost of time lost due to treatment of common adverse reactions	12.37	9.28	–25.0%
Cost of time lost due to OPV-derived polio	1,267.97	–	–100.0%
**Total**	**116.27**	**126.42**	** + 8.7%**

USD, $1 equal to S/ 3.374 Peruvian Soles (average accounting exchange rate reported on December 31, 2018 by the Peruvian Superintendence of Banks, Insurance and Private Pension Fund Administrators [Superintendencia de Banca, Seguros y AFP]) [36]. OPV, oral polio vaccine; USD, US dollar

#### Vaccination costs

The hexavalent vaccine scheme would increase direct vaccine administration costs from $60.86 to $98.66 (+ 62.1%) (Table 1). The greatest contributor to this higher cost was the mean acquisition cost of the vaccine ($20.6 [~ S/ 69.5] per dose, compared with $1.1, $5.3, and $0.1 [~ S/ 3.7, S/ 17.9, and S/ 0.4] for the pentavalent vaccine, IPV, and OPV, respectively) [24]. Therefore, the acquisition cost of vaccines to protect one infant under the current scheme was $13.98 (~ S/ 47.16), compared with $61.80 (~ S/ 208.51) using the hexavalent vaccine.

Costs varied according to where the vaccine was administered. Under the alternative scheme, if received in the EESS, the cost would be increased from $57.28 to $97.00 (+ 69.3%); if administered outside of the EESS, the cost would be reduced from $121.38 to $115.12 (–5.2%). Because this scheme only involves one vaccine (compared with three under the current), it would require less healthcare practitioner time, medical equipment and general expenses to ensure the adequate transportation of the vaccines.

#### Logistical costs

Logistical costs per dose were highest for IPV ($2.20), followed by the hexavalent ($0.78), pentavalent ($0.46), and OPV ($0.04) vaccines (Supplementary Table 4). Therefore, the logistical costs associated with protecting one infant would be lower with the alternative scheme ($2.33) compared with the current scheme ($5.80), representing a cost saving of 59.8% (Table 1).

In addition, the alternative scheme would make use of only 4.0% of warehouse space to store the hexavalent vaccine, freeing 17.5% of the total cold chain storage space required for the National Immunization Program vaccines.

#### Adverse reaction costs

The costs of treating a child who develops both local and systemic reactions and OPV-derived polio would be reduced by 99.8% under the alternative scheme, decreasing costs from $11,293.89 to $28.22 (Table 1).

The estimated cost of treating local and systemic adverse reactions in one child was $28.22 when using the alternative scheme compared with $37.75 with the current scheme (–25.3%). Although both schemes can cause the same common adverse reactions, the probability of developing these types of reactions is higher under the current scheme due to the presence of the wP component of the pentavalent vaccine (Supplementary Fig. 1). Furthermore, because the hexavalent vaccine eliminates the risk of OPV-derived polio, it would generate a cost saving of $11,256.14 per child (Table 1).

#### Social costs

In order to fully immunize their child and resolve associated complications, assuming the scenario that a child develops both types of adverse reactions, parents would spend ~ 935 h less under the alternative scheme. This accounts for a reduction of 0.8 h for vaccination activities, 2.3 h for follow-up and treatment of local and systemic adverse reactions, and 932.4 h for polio diagnosis and subsequent treatment. This would result in savings of 98.6% per child vaccinated ($17.50 with the hexavalent vaccine vs $1,289.65 under the current scheme) (Table 1).

When expanding this estimate for the entire target population, an overall reduction of 1,759,042 h with the alternative scheme was calculated. Accordingly, the estimated total cost was $7,463,891 with the hexavalent vaccine versus $9,854,066 with the current scheme (–24.3%). This value included a 15.7% reduction in the cost of time ($3,593,529 vs $4,263,510), because of fewer vaccination appointments and fewer vaccines administered at each appointment (Supplementary Tables 5 and 6). It also accounts for a 30.7% reduction in the cost of time linked with treatment of local and systemic adverse reactions ($3,870,361 vs $5,587,264) (Supplementary Table 7), and a 100% reduction in the cost of time to treat OPV-derived polio (no cost vs $3,291) (Supplementary Table 8).

### Budget impact analysis

**Fig. 1:**
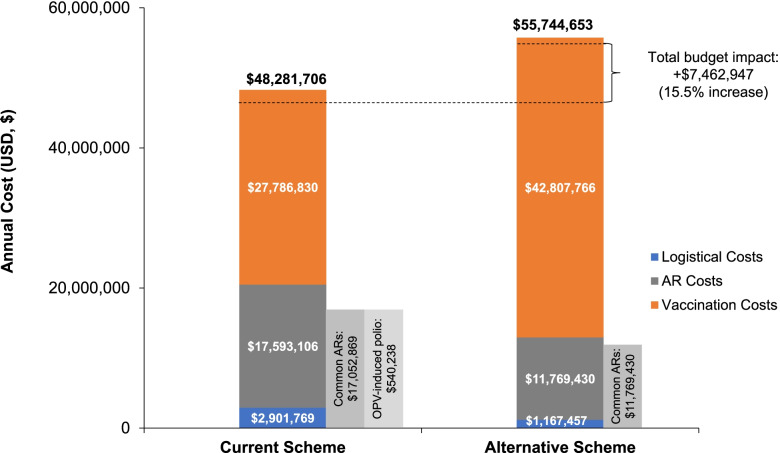
Estimated total annual cost (USD, $) associated with vaccination, according to cost category. USD, $1 equal to S/ 3.374 Peruvian Soles (average accounting exchange rate reported on December 31, 2018 by the Peruvian Superintendence of Banks, Insurance and Private Pension Fund Administrators [Superintendencia de Banca, Seguros y AFP]) [36]. ARs, adverse reactions; OPV, oral polio vaccine; USD, US dollar

The estimated total annual cost to finance the implementation of vaccination activities nationwide under the current scheme was $48,281,706, compared with $55,744,653 for the alternative scheme (Fig. 1). This represents a budgetary impact of + $7,462,947 (+ 15.5%) with the alternative scheme, mainly associated with the larger direct vaccine administration costs. Substituting the current scheme with the alternative scheme would increase total annual costs associated with vaccination by $15,020,936 (54.1%), from $27,786,830 to $42,807,766 (Fig. 1; Supplementary Tables 2 and 3).

Conversely, the total annual cost to guarantee the vaccine supply chain until its arrival at the healthcare facility would be lower with the hexavalent vaccine. The estimated logistical cost per annum was $2,901,769 for the current scheme, compared with $1,167,457 for the alternative scheme (Fig. 1), for an annual cost saving of 59.8%.

Similarly, the estimated total annual cost of treating all cases of adverse reactions was 33.1% lower with the alternative scheme ($11,769,430) compared with the current scheme ($17,593,106) (Fig. 1).

Specifically, the estimated total annual cost of treating local and systemic adverse reactions was $11,769,430 with the hexavalent vaccine compared with $17,052,869 under the current scheme, representing a cost saving of 31.0% (Supplementary Table 9). A lower number of vaccinated infants would experience ≥ 4 local and/or systemic adverse reactions with the hexavalent vaccine (83,192) compared with the current scheme (313,796). Consequently, the cost of treating these infants with ≥ 4 events would be reduced by 74% with the alternative scheme compared with the current scheme ($3,425,919 versus $13,161,210, respectively). The use of the hexavalent vaccine would avoid all costs associated with OPV-derived polio (estimated at $540,238 per annum under the current scheme) (Supplementary Table 10).

### Sensitivity analysis

**Fig. 2:**
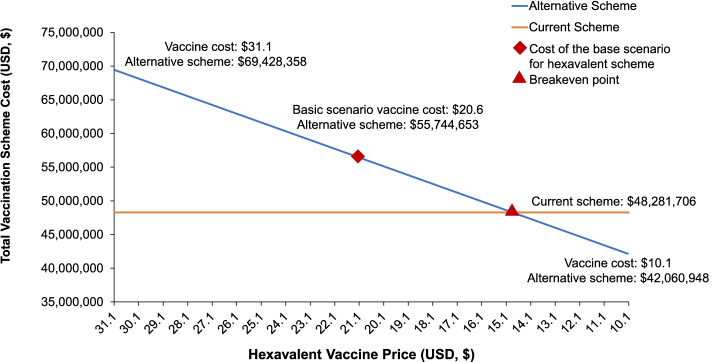
One way sensitive analysis. Variation in estimated total annual costs for the payer (USD, $) when modifying the hexavalent vaccine acquisition cost per dose. USD, $1 equal to S/ 3.374 Peruvian Soles (average accounting exchange rate reported on December 31, 2018 by the Peruvian Superintendence of Banks, Insurance and Private Pension Fund Administrators [Superintendencia de Banca, Seguros y AFP]).[36]. USD, US dollar

Of the scenarios analyzed, only those involving a variation in the hexavalent vaccine acquisition cost showed a significant impact on the total cost of the alternative scheme. Varying only the hexavalent vaccine price per dose resulted in a total annual cost reduction to $42,060,948, when the cost dropped by 49% ($10.1 per dose); and an increase to $69,428,358, when the cost rose by 51% ($31.1 per dose). The breakeven price is ~ $14.87 per hexavalent dose (Fig. 2). Below this price, introducing the alternative scheme represents a financially feasible option from the payer perspective. Variations in case volume and vial weight did not result in considerable changes in total costs estimated in the basic scenario.

## Discussion

CMA and BIA analyses of replacing the current scheme with a hexavalent vaccine resulted in a net increase in costs of 8.7% and 15.5%, respectively. This was primarily driven by the higher acquisition cost per dose of the hexavalent vaccine compared with the other three vaccines, which impacted considerably the healthcare costs to administer the vaccine. When analyzing other activities related to the immunization process, savings were found on logistics costs and costs associated with treatment of adverse reactions.

Compared with the CMA, the BIA estimated a smaller reduction in costs related to treatment of adverse reactions in the alternative scheme versus the current scheme. This is likely because of the low risk of developing vaccine-derived polio across the entire population, minimizing the budget impact of this costly adverse reaction. The CMA also considered the broader perspective of social costs, showing that the alternative scheme would result in an overall saving of time parents spent on medical appointments to immunize their child and treating any adverse reaction, translating into a total cost saving of 24.3%. In this sense, it would be of interest to analyze other indirect impacts of substituting the current scheme with the hexavalent vaccine.

### Logistics

Replacement of the current vaccination scheme with the hexavalent vaccine was found to result in a logistical cost saving of 59.8% (equivalent in total to more than $1.7 million), as well as freeing of ~ 18.0% of the required Peruvian vaccines storage capabilities. This additional benefit is associated with the smaller volume and weight of the hexavalent vaccine compared with the pentavalent vaccine, IPV, and OPV combined.

Freeing transportation and storage space would increase the efficiency of the supply chain and avoid bottlenecks caused by an insufficient capacity to receive supplies at a national and local level [40].

The smaller hexavalent vaccine dimensions could potentially generate savings during vaccination campaigns outside healthcare facilities, particularly in rural areas where cold storage space may be more limited and vaccination campaigns more frequent because of geographic dispersion.

### Local and systemic adverse reactions

As reflected by the 33.1% lower adverse reaction costs (equivalent in total to ~ $5.8 million), the alternative scheme is expected to be associated with fewer events than the current scheme. In terms of local and systemic reactions, this effect is largely due to the aP component of the hexavalent vaccine, [17, 41] which replaces the wP component included in the pentavalent vaccine of the current scheme [2]. wP is more reactogenic than aP, and has a higher incidence of injection site and systemic adverse reactions, such as fever, erythema, swelling, and drowsiness [42, 43]. A previous study carried out in Peru found that the hexavalent vaccine results in 20% fewer episodes of fever > 38 °C and a considerably lower incidence of local and systemic reactions than a vaccination scheme based on a pentavalent vaccine and OPV [43].

### Vaccine-derived polio

A further contributor to the reduced adverse reaction costs associated with the hexavalent vaccine is the elimination of the risk of OPV-derived polio. Although rare, [44] acute paralytic polio is an extremely serious and debilitating disease, with no cure and mortality rates of 5–10% [8]. The last recorded case of wild poliovirus-derived disease in the Americas occurred in 1991 [44]. However, in Peru, 81 cases of acute flaccid paralysis were investigated in 2011, with three considered acute paralytic polio [44]. In 2017, according to the MINSA National Centre of Disease Epidemiology, Prevention and Control, 55 cases of suspected acute flaccid paralysis were notified in Peru [45].

Given that identifying, monitoring, and confirming suspected polio cases, as well as treating confirmed cases, entail a substantial cost to the Peruvian Government (estimated as ~ $0.5 million [S/ 1.8 million] per annum in the current study), the ability of the hexavalent vaccine to replace OPV and, thereby, eradicate the risk of vaccine-associated polio, is a substantial benefit; particularly, if it is considered the burden of disease that could be prevented. Prior studies have documented that, for upper-middle income countries (as Peru has been classified by the World Bank), 14 disability-adjusted life years (DALY) are lost per VAPP case [46, 47]. Although, country-specific estimations varied across regions. In Shanghai, it was estimated that switching from a four trivalent oral polio vaccine (tOPV) schedule to a four-IPV schedule could prevent 1.35 VAPP cases and 18.96 DALYs annually [48]. Whereas, a study in Colombia found that using OPV could cause between 2 and 4 VAPP cases during the two years of follow-up of the study and, consequently, introducing IPV could avoid 64 DALYs [49]. Given its sociodemographic similarities, the last study may better reflect the burden of disease averted in Peru; though it is still of high interest for future research to estimate the health benefits of adopting and IPV-containing schedule, that considers country-specific epidemiological and vaccination coverage data.

Moreover, not only could OPV cause isolated paralytic polio cases, but it also has the potential to cause cases of cVDPV, developing polio outbreaks in areas previously free of the disease [50]. For this reason, since the Polio Eradication and Endgame Strategic Plan 2013–2018 was elaborated by the Global Polio Eradication Initiative (GPEI) and approved by the WHO Executive Board, efforts towards a phased removal of all types of OPVs have been taken place globally [51, 52]. In 2016, a coordinated switch from the tOPV to the bivalent oral polio vaccine (bOPV) was implemented, preceded by the introduction of at least one dose of IPV vaccine in national immunization programs; with the aim to reduce the risk of OPV-derived polio cases associated with the type 2 component of the tOPV vaccine. However, the risk of type 2 polio outbreaks is growing (64 type 2 cVDPV outbreaks have been reported since the switch through 2020, affecting 33 countries) and there is still the risk to develop type 1 and 3 cVDPV cases. Thus, in its last updated Polio Eradication Strategic Plan 2022–2026, the goal to complete the phase out of all OPV vaccines and the transition to IPV exclusive use was reinforced, whether as a standalone vaccine or as part of a combination vaccine [20]. Likewise, the Latin American Society for Pediatric Infectious Disease (Sociedad Latinoamericana de Infectología Pediátrica) recommends a regional transition away from OPV and towards IPV [53] and many national healthcare services have already discontinued use of OPV altogether, including the US, Uruguay, and Chile [12, 54, 55]. Including the hexavalent vaccine in the National Immunization Program of Peru would be an option to achieve this objective.

### Efficiency

In addition to the aforementioned cost advantages, reducing adverse reaction rates and eliminating OPV-derived polio by use of the alternative scheme would decrease the amount of time a healthcare professional must dedicate to solve adverse reactions, as well as time spent by parents caring for a sick child.

Furthermore, as a combination vaccine, the hexavalent vaccine could reduce the risk of vaccination sequence disruption and delayed vaccination, contributing to improved vaccination coverage. Under the current scenario, according to the Endes 2017, there is a fraction of children under one year of age who only complete the primary polio vaccination schedule but not the primary pentavalent scheme (~ 3%), and vice versa (~ 2%). By using a more combine scheme, these children lost to follow-up could have been fully protected, increasing the vaccination coverage rate for both vaccines (i.e., children who received three doses of pentavalent and polio vaccine) from 75.2% to ~ 80% [56]. However, more integrated vaccination schemes may be more sensitive to supply interruptions, negatively affecting vaccination coverage. Hence, a balance between both effects should be considered.

Finally, it is important to mention that there are other alternative vaccination schemes that the country could adopt which could mitigate the risk of developing adverse reactions such as the replacement of the existing third dose of OPV with the IPV vaccine or the introduction of a pentavalent acellular vaccine instead of a whole cellular one. Even if these alternatives could be less costly in terms of vaccine administration (as the vaccine acquisition cost may be lower), they could represent a higher logistic cost and a greater complexity to be implemented due to a greater storage space requirement. Also, the efficiency gain and the social benefits that brings more integrated vaccination schemes would be lose. All of these factors should be evaluated when deciding to transition to a new immunization scheme.

### Limitations

From a payer perspective, CMAs and BIAs are essential for the comprehensive economic assessment of a novel healthcare intervention, and are important evidence for reimbursement authorities [39]. Nevertheless, while conducted in line with ISPOR recommendations using the most appropriate data sources available, the present analysis was based on a number of assumptions and results should be interpreted as best estimates of real-world outcomes. For example, the vaccine uptake rates for the hexavalent vaccines and the current scheme were assumed to be the same; however, this may not be representative of clinical practice and requires further investigation. In addition, the study had a limited time horizon of 1 year and only considered vaccine effects in infants < 1 year of age; thus, any long-term effects (such as long-term costs associated with polio rehabilitation and the long-term economic return of disease prevention at a population level) were not captured.

It should also be noted that BIAs are not intended to capture aspects such as productivity and costs outside of the healthcare system [39]. Although this study incorporated the differences in cost of time society would assume under both scenarios, it does not recognize other intangible impacts, such as the potential effects on the utilization of human and physical resources.

## Conclusions

According to the present study, including the hexavalent vaccine into the National Immunization Program of Peru in place of the current vaccination scheme for infants < 1 year of age would increase the public financial budget of the government by 15.5%. Despite the significant reduction of logistical and adverse reaction costs, it would represent larger vaccine administration costs.

Incorporating the indirect costs associated with time spent by parents to complete the immunization program would reduce the budgetary impact to 8.7% with the use of the hexavalent vaccine, thereby demonstrating the social value of the alternative scheme. This merits consideration by government bodies, and future studies investigating such benefits would be informative.

## Supplementary Information

**Additional file 1.**

## Data Availability

All data generated or analyzed during this study are included in this published article [and its supplementary information files].

## Abbreviations

aP: Acellular pertussis
BIA: Budgetary impact analysis
bOPV: Bivalent oral polio vaccine
CMA: Cost-minimization analysis
cVDPV: Circulating vaccine-derived poliovirus
DALY: Disability-adjusted life years
EESS: Establecimientos de salud
GPEI: Global Polio Eradication Initiative
IPV: Inactivated polio vaccine
ISPOR: International Society for Pharmacoeconomics and Outcomes Research
Hib: *Haemophilus influenzae* type B
MINSA: Ministerio de Salud del Perú
OPV: Oral polio vaccine
tOPV: Trivalent oral polio vaccine
VAPP: Vaccine-associated paralytic poliomyelitis
WHO: World Health Organization
wP: Whole cell pertussis
