# Supplementary material for: Economic assessment of incorporating the hexavalent vaccine as part of the National Immunization Program of Peru
Source: BMC Health Serv Res. 2022 May 16;22:651. doi: 10.1186/s12913-022-08006-1 (PMC9109284; doi:10.1186/s12913-022-08006-1)
Supplement: Supplementary file 1 — Additional file 1. [file 12913_2022_8006_MOESM1_ESM.docx]

Economic assessment of incorporating the Hexavalent vaccine as part of the National Immunization Program of Peru

Supplementary methods

Logistical costs

All costs from national planning and vaccine acquisition to national, regional, and local storage and distribution to the EESS were considered “logistical costs”. These were calculated on an annual basis, taking into account five different geographical areas: Lima metropolitan, urban cities outside of Lima, rural coastal, rural mountain, and rural jungle. The logistical cost for each area was approximated using the logistical chain of an EESS in each region as reference, with individual costs estimated for each of the following stages of the supply chain.

For planning and acquisition, the model included human resource costs, office equipment and supplies, travel and daily subsistence expenses, based on administrative records and current regulations.^1-5^ For distribution costs from the national to regional warehouses, the weight per dose of each vaccine container was accounted (16.179 g, hexavalent vaccine; 16.179 g, pentavalent vaccine; 46.876 g, IPV; 1.223 g, OPV) using import records between 2016 and 2018 collected in the Veritrade database. For storage costs, the volume per dose of each vaccine container was used (11.319 cm^3^, hexavalent vaccine; 14.060 cm^3^, pentavalent vaccine; 68.900 cm^3^, inactivated polio vaccine [IPV]; 0.879 cm^3^, oral polio vaccine [OPV]). The official measures were obtained from the Pan American Health Organization (PAHO)/WHO website, according to the local provider and vaccine presentation used in the country, which was identified based on the import records from 2016 to 2018 reported in the Veritrade database. In the case of the IPV vaccine, official measures provided by Sanofi Pasteur were used as it is the local supplier and there was no public information about its measures under the presentation that is acquired in Peru.

Data on the target population in each area were extracted from administrative records published on the websites of the corresponding Regional Healthcare Directorate or Administration (Geresa, Diresa, or Diris).^6-9^

Vaccination costs

Costs associated with the vaccination process were estimated based on methodology and guidelines defined by MINSA in 2009.^10^ This involved a bottom-up costing approach allowing cost estimation for each related medical procedure, assuming technical efficiency. Personnel expenses; medical equipment, devices and supplies; infrastructure; medical support services; and administrative and general expenses were considered.

Cost estimates considered possible variations in the dosing schedule.^11^ Twenty-one immunization variants were identified for the current vaccination scheme (Supplementary Table 2) and three for the alternative vaccination scheme (Supplementary Table 3).

Adverse reaction costs

Adverse reaction costs were calculated using a bottom-up costing approach.^10^ Two groups of adverse reactions were considered: local and systemic (both schemes); and OPV-derived polio (current scheme). The probability of developing local and systemic adverse reactions were determined using the results of a 2018 systematic audit. Rates in infants receiving a vaccine containing acellular pertussis (aP; similar to that in the hexavalent vaccine) were compared with those in infants receiving a vaccine containing the full pertussis cell (wP; similar to that in the pentavalent vaccine).^12^ Taking these rates into consideration, a Monte Carlo simulation with 50,000 iterations was executed to estimate the probability of adverse reactions using each of the two schemes (Supplementary Figure 1). Variation in event presentation was also taken into account (Supplementary Table 9).

The costs associated with identifying suspected OPV-derived polio cases, their subsequent monitoring and confirmation were also calculated based on acute flaccid paralysis epidemiological surveillance guideline (Directive N° 059-Minsa/DGE-V-01) approved by Minsa in 2015. Moreover, the treatment of polio confirmed cases were estimated using a bottom-up costing approach, taking into account the related medical procedures reported by specialist physicians.^13^ OPV-derived polio rates were based on the figures reported by the MINSA National Centre of Disease Epidemiology, Prevention and Control.^14^

Social costs

Social costs were calculated using the “social value of time” specified by the Ministry of Economics and Finance in the Directive 002-2017EF/63.01.^15^ It was assumed that only one parent accompanied their child to the appointment using the appropriate local or intercity public transport for the geographical area, and did not perform productive activities during that time.

Given that vaccination and treatment for local and systemic adverse reactions usually take place at primary healthcare facilities and that these are generally located within the patient’s locality, the social value of time for urban public transportation was used to estimate costs associated with these visits. OPV-derived polio requires more specialized treatment at tertiary healthcare facilities, which are usually only available in large cities. Accordingly, the social value of time for public interurban transportation was used for these visits. The average time parents spent traveling and waiting to be attended were estimated based on the National Household Survey 2017.^16^ The average time spent receiving medical attention was based on specialist physicians communications.

Sensitivity analysis

A sensitivity analysis was carried out to establish the effect of modifying key variables relating to hexavalent pricing and presentation on total cost estimates in the BIA. In the first scenario the hexavalent vaccine acquisition cost was assumed to vary from 49% to 151% of values used in the basic case. In the second scenario, the case volume variation was simulated to vary from 60% to 120%. In the third scenario, the vial weight was assumed to vary from 60% to 140%. Finally, the fourth scenario assumed a variation in the hexavalent vaccine acquisition cost and case volume; the range for each parameter was the same as described for scenarios one and two.

Supplementary tables

Supplementary Table 1. Summary of vaccine characteristics

|  | **Current scheme** | | | **Alternative scheme** |
| --- | --- | --- | --- | --- |
| **Vaccine** | **Pentavalent vaccine  (DTwP-HB-Hib)^17^** | **IPV^17^** | **bOPV^17^** | **Hexavalent vaccine (DTaP-HB-Hib-IPV)^18,19^** |
| Indications | Prevention of diphtheria, tetanus, pertussis, hepatitis B, and diseases caused by Hib | Prevention of poliomyelitis types 1, 2, and 3 | Prevention of poliomyelitis types 1 and 3 | Prevention of diphtheria, tetanus, pertussis, hepatitis B, poliomyelitis, and diseases caused by Hib |
| Composition per dose | - Diphtheria toxoid 25 Lf (30 IU)   - - - Tetanus toxoid 5 Lf (40 IU)       - *Bordetella pertussis* (inactivated whole cell) 16 OU (4 IU)       - HbsAg (rDNA) 10 μg - Adsorbed on aluminum phosphate, Al^3+^ 1.25 mg - Thiomersal 0.005% | - IPV: - Type 1 (Mahoney) 40 U antigen D - Type 2 (MEF-1) 8 U antigen D - Type 3 (Saukett) 32 U antigen D   - - - Excipients | - Polio virus (Sabin) grown in primary culture in monkey kidney   - - - Type 1 ≥10^6.0^ CCID_50_       - Type 3 ≥10^5.8^ CCID_50_       - Neomycin 15 μg - Stabilizer 1 M MgCl_2_ | - Diphtheria toxoid ≥20 μL   - - - Tetanus toxoid ≥40 μL       - *Bordetella pertussis* antigens:   - Pertussis toxoid 25 μg   - Filamentous haemagglutinin 25 μg - IPV: - Type 1 (Mahoney) 40 U antigen D - Type 2 (MEF-1) 8 U antigen D - Type 3 (Saukett) 32 U antigen D   - - - HbsAg 10 μg* - *Hib* polysaccharide 12 μg   - - - Conjugated with tetanus protein  22–36 μg |
| Presentation | Cardboard box containing 50 glass ampoules or vials (single doses) | Cardboard box containing 1 preloaded, colorless glass syringe with 25G × 5.8” needle (single doses) | Cardboard box containing 50 oral suspension in multidose bottles (20 doses per bottle) | Cardboard box containing 10 glass ampoules or vials (single doses) |
| Storage temperatures | +2°C to +8°C | +2°C to +8°C | +2°C to +8°C | +2°C to +8°C |
| Duration once opened | Immediate use | Immediate use | Up to 4 weeks | Immediate use |
| Administration route | Intramuscular^†^ | Intramuscular^‡^ | Oral^§^ | Intramuscular^II^ |
| Volume per dose | 0.5 mL | 0.5 mL | Two drops | 0.5 mL |
| Wastage factor | 0 (0%) | 0 (0%) | 1.3 (30%) | 0 (0%) |

*B5 antigen; ^†^one intramuscular dose each at 2, 4, and 6 months; ^‡^one intramuscular dose each at 2 and 4 months; ^§^one oral dose at 6 months; ^II^one intramuscular dose at 2, 4, and 6 months

bOPV, bivalent oral polio vaccine; CCID50, cell culture infectious dose 50%; DtaP, diphtheria, tetanus and acellular pertussis; DTwP, diphtheria tetanus whole cell pertussis; HB, hepatitis B; HbsAg, hepatitis B virus surface antigen; Hib, *Haemophilus* *influenzae* type B; IPV, inactivated polio vaccine; IU, international units; Lf, limits of flocculation; MEF-1, mouse embryonic fibroblast-1; MgCl2, magnesium chloride; OU, opacity units; rDNA, recombinant deoxyribonucleic acid; U, units; USD, US dollar.

Supplementary Table 2. Estimated total annual costs of vaccine administration for the current scheme (USD, $), by variation in the immunization schedule

| **Scheme completion** | **Level of integration*** | **Number of doses administered** | **Cost (USD,** **$)** |
| --- | --- | --- | --- |
| No | Fully integrated | 1 pentavalent; 1 polio | 299,174 |
|  |  | 2 pentavalent; 2 polio | 535,337 |
|  | Non-integrated | 1 pentavalent; 1 polio | 844 |
|  |  | 1 pentavalent; 2 polio | 2,356 |
|  |  | 2 pentavalent; 1 polio | 1,404 |
|  |  | 3 pentavalent; 1 polio | 56 |
|  |  | 2 pentavalent; 3 polio | 573 |
|  |  | 3 pentavalent; 2 polio | 363 |
|  | Partially integrated | Integrated: 1 pentavalent; 1 polio  Separate: 1 pentavalent; 1 polio | 124,961 |
|  |  | Integrated: 1 pentavalent; 1 polio Separate: 1 pentavalent; 2 polio | 683 |
|  |  | Integrated: 1 pentavalent; 1 polio Separate: 2 pentavalent; 1 polio | 702 |
|  |  | Integrated: 1 pentavalent; 1 polio  Separate: 1 pentavalent | 26,095 |
|  |  | Integrated: 1 pentavalent; 1 polio  Separate: 2 pentavalent | 47,340 |
|  |  | Integrated: 1 pentavalent; 1 polio  Separate: 1 polio | 34,816 |
|  |  | Integrated: 1 pentavalent; 1 polio  Separate: 2 polio | 70,817 |
|  |  | Integrated: 2 pentavalent; 2 polio  Separate: 1 pentavalent | 1,889,813 |
|  |  | Integrated: 2 pentavalent; 2 polio  Separate: 1 polio | 2,189,649 |
| Yes | Fully integrated | 3 pentavalent; 3 polio | 21,812,018 |
|  | Non-integrated | 3 pentavalent; 3 polio | 720,548 |
|  | Partially integrated | Integrated: 2 pentavalent; 2 polio  Separately: 1 pentavalent; 1 polio | 11,479 |
|  |  | Integrated: 1 pentavalent; 1 polio  Separately: 2 pentavalent; 2 polio | 17,799 |
| **Total:** | | | **27,786,830** |

USD, $1 equal to S/ 3.374 Peruvian Soles (average accounting exchange rate reported on December 31, 2018 by the Peruvian Superintendence of Banks, Insurance and Private Pension Fund Administrators [Superintendencia de Banca, Seguros y AFP]).^20^

*“Fully integrated” refers to the two vaccines being administered during the same visit in all cases, “non-integrated” to vaccines being administered at separate visits in all cases, and “partially integrated” to a mixture of the two.

USD, US dollar.

Supplementary Table 3. Estimated total annual costs of vaccine administration for the alternative scheme (USD, $), by variation in the immunization schedule

| **Scheme completion** | **Number of doses administered** | **Cost (USD, $)** |
| --- | --- | --- |
| No | 1 | 574,518 |
|  | 2 | 5,660,267 |
| Yes | 3 | 36,572,981 |
| **Total:** | | **42,807,766** |

USD, $1 equal to S/ 3.374 Peruvian Soles (average accounting exchange rate reported on December 31, 2018 by the Peruvian Superintendence of Banks, Insurance and Private Pension Fund Administrators [Superintendencia de Banca, Seguros y AFP]).^20^

USD, US dollar.

Supplementary Table 4. Estimated national logistical cost per dose of vaccine (USD, $)

|  | **Cost per dose (USD, $)** | | | |
| --- | --- | --- | --- | --- |
| **Logistical stage** | **Pentavalent vaccine** | **IPV** | **OPV** | **Hexavalent vaccine** |
| Planning | 0.0011 | 0.0016 | 0.0032 | 0.0011 |
| Acquisition | 0.0004 | 0.0005 | 0.0011 | 0.0004 |
| Transport to national warehouse | 0.0034 | 0.0099 | 0.0003 | 0.0034 |
| National warehouse | 0.0042 | 0.0207 | 0.0003 | 0.0034 |
| Transport to regional warehouse | 0.0155 | 0.0448 | 0.0015 | 0.0155 |
| Regional warehouse | 0.0044 | 0.0217 | 0.0004 | 0.0036 |
| Network warehouse | 0.4131 | 2.0242 | 0.0336 | 0.7391 |
| Micro-network warehouse | 0.0148 | 0.0723 | 0.0012 | 0.0119 |
| **Total:** | **0.4568** | **2.1958** | **0.0415** | **0.7783** |

USD, $1 equal to S/ 3.374 Peruvian Soles (average accounting exchange rate reported on December 31, 2018 by the Peruvian Superintendence of Banks, Insurance and Private Pension Fund Administrators [Superintendencia de Banca, Seguros y AFP]).^20^

IPV, inactivated polio vaccine; OPV, oral polio vaccine; USD, US dollar.

Supplementary Table 5. Estimated total annual costs (USD, $) of time lost due to the vaccine administration process under the current scheme

| **Scheme completion** | **Level of integration*** | **Number of doses administered** | **Cost of time (USD, $)^†^** | **Number of cases** | **Total cost (USD, $)** |
| --- | --- | --- | --- | --- | --- |
| No | Fully integrated | 1 pentavalent; 1 polio | 1.7 | 13,511 | 40,873 |
|  |  | 2 pentavalent; 2 polio | 3.5 | 12,074 | 72,970 |
|  | Non-integrated | 1 pentavalent; 1 polio | 4.6 | 28 | 165 |
|  |  | 1 pentavalent; 2 polio | 6.2 | 49 | 418 |
|  |  | 2 pentavalent; 1 polio | 7.6 | 32 | 271 |
|  |  | 3 pentavalent; 1 polio | 12.0 | 1 | 12 |
|  |  | 2 pentavalent; 3 polio | 13.6 | 8 | 117 |
|  |  | 3 pentavalent; 2 polio | 15.0 | 5 | 75 |
|  | Partially integrated | Integrated: 1 pentavalent; 1 polio  Separate: 1 pentavalent; 1 polio | 5.5 | 2,373 | 20,808 |
|  |  | Integrated: 1 pentavalent; 1 polio Separate: 1 pentavalent; 2 polio | 12.2 | 11 | 134 |
|  |  | Integrated: 1 pentavalent; 1 polio Separate: 2 pentavalent; 1 polio | 12.2 | 11 | 134 |
|  |  | Integrated: 1 pentavalent; 1 polio  Separate: 1 pentavalent | 4.3 | 748 | 4,449 |
|  |  | Integrated: 1 pentavalent; 1 polio  Separate: 2 pentavalent | 5.8 | 970 | 8,388 |
|  |  | Integrated: 1 pentavalent; 1 polio  Separate: 1 polio | 3.3 | 879 | 5,168 |
|  |  | Integrated: 1 pentavalent; 1 polio  Separate: 2 polio | 5.8 | 1,394 | 12,254 |
|  |  | Integrated: 2 pentavalent; 2 polio  Separate: 1 pentavalent | 5.1 | 32,903 | 293,033 |
|  |  | Integrated: 2 pentavalent; 2 polio  Separate: 1 polio | 5.1 | 39,352 | 352,449 |
| Yes | Fully integrated | 3 pentavalent; 3 polio | 5.3 | 361,886 | 3,301,693 |
|  | Non-integrated | 3 pentavalent; 3 polio | 11.9 | 8,423 | 144,755 |
|  | Partially integrated | Integrated: 2 pentavalent; 2 polio  Separately: 1 pentavalent; 1 polio | 9.5 | 168 | 1,990 |
|  |  | Integrated: 1 pentavalent; 1 polio  Separately: 2 pentavalent; 2 polio | 11.3 | 231 | 3,353 |
| **Total:** | | | **161.3** | **475,057** | **4,263,510** |

USD, $1 equal to S/ 3.374 Peruvian Soles (average accounting exchange rate reported on December 31, 2018 by the Peruvian Superintendence of Banks, Insurance and Private Pension Fund Administrators [Superintendencia de Banca, Seguros y AFP]).^20^

*“Fully integrated” refers to the two vaccines being administered during the same visit in all cases, “non-integrated” to vaccines being administered at separate visits in all cases, and “partially integrated” to a mixture of the two. ^†^The average time cost for each of the sub-variants that form part of the clinical variant is given. Sub-variants represent the place where each vaccine dose is administered, within or outside the EESS.
EESS, establecimientos de salud; USD, US dollar.

Supplementary Table 6. Estimated total annual costs (USD, $) of time lost due to the vaccine administration process under the alternative scheme

| **Scheme completion** | **Number of doses administered** | **Cost of time (USD, $)*** | **Number of cases** | **Total cost (USD, $)** |
| --- | --- | --- | --- | --- |
| No | 1 | 1.6 | 17,612 | 50,443 |
|  | 2 | 3.2 | 86,737 | 496,192 |
| Yes | 3 | 4.7 | 370,708 | 3,046,895 |
| **Total:** | | **9** | **475,057** | **3,593,529** |

USD, $1 equal to S/ 3.374 Peruvian Soles (average accounting exchange rate reported on December 31, 2018 by the Peruvian Superintendence of Banks, Insurance and Private Pension Fund Administrators [Superintendencia de Banca, Seguros y AFP]).^20^

*The average time cost for each of the sub-variants that form part of the clinical variant is given. Sub-variants represent the place where each vaccine dose is administered, within or outside the EESS.

EESS, establecimientos de salud; USD, US dollar.

Supplementary Table 7. Estimated total annual costs (USD, $) of time lost due to local and systemic adverse reactions for the current and alternative schemes

|  |  | **Current scheme** | | **Alternative scheme** | |
| --- | --- | --- | --- | --- | --- |
| **Number of ARs** | **Type of AR** | **Number of cases** | **Total cost (USD, $)** | **Number of cases** | **Total cost (USD, $)** |
| 1 | 1 local | 3,402 | 8,860 | 25,264 | 65,794 |
|  | 1 systemic | 3,696 | 19,251 | 49,093 | 255,700 |
| 2 | 2 local | 5,807 | 30,246 | 13,129 | 68,382 |
|  | 2 systemic | 8,401 | 87,513 | 48,764 | 507,974 |
|  | 1 local and 1 systemic (same dose) | 10,585 | 55,132 | 38,404 | 200,027 |
|  | 1 local and 1 systemic (different doses) | 10,284 | 80,346 | 36,980 | 288,915 |
| 3 | 3 local | 3,717 | 29,040 | 1,632 | 12,750 |
|  | 3 systemic | 5,903 | 92,237 | 13,887 | 216,991 |
|  | 2 local and 1 systemic (two from the same dose) | 19,884 | 155,348 | 19,689 | 153,825 |
|  | 2 local and 1 systemic (different doses) | 19,292 | 200,964 | 15,956 | 166,213 |
|  | 1 local and 2 systemic (two from the same dose) | 23,844 | 248,382 | 39,295 | 409,335 |
|  | 1 local and 2 systemic (different doses) | 23,135 | 301,246 | 31,844 | 414,648 |
| 4 | 3 local and 1 systemic | 25,168 | 262,175 | 5,220 | 54,377 |
|  | 2 local and 2 systemic (all from the same dose) | 44,780 | 466,472 | 20,302 | 211,486 |
|  | 2 local and 2 systemic (two from the same dose) | 43,448 | 565,746 | 16,453 | 214,238 |
|  | 1 local and 3 systemic | 35,074 | 548,048 | 22,465 | 351,026 |
| 5 | 2 local and 3 systemic | 66,098 | 1,032,812 | 11,381 | 177,833 |
|  | 3 local and 2 systemic | 56,461 | 735,191 | 5,502 | 71,643 |
| 6 | 3 local and 3 systemic | 42,767 | 668,254 | 1,869 | 29,204 |
| **Total:** | | **451,746** | **5,587,264** | **417,129** | **3,870,361** |

USD, $1 equal to S/ 3.374 Peruvian Soles (average accounting exchange rate reported on December 31, 2018 by the Peruvian Superintendence of Banks, Insurance and Private Pension Fund Administrators [Superintendencia de Banca, Seguros y AFP]).^20^

AR, adverse reaction; USD, US dollar.

Supplementary Table 8. Estimated total annual costs (USD, $) of time lost due to OPV-induced post-vaccination polio for the current scheme

| **Variant** | **Cost of time (USD, $)** | **Number of cases** | **Total cost (USD, $)** |
| --- | --- | --- | --- |
| Diagnosis and monitoring of suspected AFP cases | 37 | 55 | 2,061 |
| Confirmed polio case – Paraplegia with preserved sensitivity | 956 | 1 | 956 |
| Confirmed polio case – Postural disorders or postural scoliosis | 274 | 1 | 274 |
| **Total:** | | | **3,291** |

USD, $1 equal to S/ 3.374 Peruvian Soles (average accounting exchange rate reported on December 31, 2018 by the Peruvian Superintendence of Banks, Insurance and Private Pension Fund Administrators [Superintendencia de Banca, Seguros y AFP]).^20^

AFP, acute flaccid paralysis; OPV, oral polio vaccine; USD, US dollar.

Supplementary Table 9. Estimated total annual costs (USD, $) of treating adverse reactions for the current and alternative vaccination scheme

|  |  |  | **Current scheme** | | **Alternative scheme** | |
| --- | --- | --- | --- | --- | --- | --- |
| **Number of ARs** | **Type of AR** | **Variant cost (USD, $)** | **Number of cases** | **Total cost (USD, $)** | **Number of cases** | **Total cost (USD, $)** |
| 1 | 1 local | 6.3 | 3,402 | 21,531 | 25,264 | 159,892 |
|  | 1 systemic | 16.4 | 3,696 | 60,466 | 49,093 | 803,150 |
| 2 | 2 local | 12.7 | 5,807 | 73,503 | 13,129 | 166,183 |
|  | 2 systemic | 32.7 | 8,401 | 274,877 | 48,764 | 1,595,536 |
|  | 1 local and 1 systemic (same dose) | 16.4 | 10,585 | 173,168 | 38,404 | 628,281 |
|  | 1 local and 1 systemic (different doses) | 22.7 | 10,284 | 233,330 | 36,980 | 839,025 |
| 3 | 3 local | 19.0 | 3,717 | 70,573 | 1,632 | 30,986 |
|  | 3 systemic | 49.1 | 5,903 | 289,715 | 13,887 | 681,565 |
|  | 2 local and 1 systemic (two from the same dose) | 22.7 | 19,884 | 451,141 | 19,689 | 446,716 |
|  | 2 local and 1 systemic (different doses) | 29.0 | 19,292 | 559,805 | 15,956 | 463,003 |
|  | 1 local and 2 systemic (two from the same dose) | 32.7 | 23,844 | 780,165 | 39,295 | 1,285,715 |
|  | 1 local and 2 systemic (different doses) | 39.0 | 23,135 | 903,385 | 31,844 | 1,243,457 |
| 4 | 3 local and 1 systemic | 29.0 | 25,168 | 730,312 | 5,220 | 151,471 |
|  | 2 local and 2 systemic (all from the same dose) | 32.7 | 44,780 | 1,465,182 | 20,302 | 664,272 |
|  | 2 local and 2 systemic (two from the same dose) | 39.0 | 43,448 | 1,696,575 | 16,453 | 642,463 |
|  | 1 local and 3 systemic | 49.1 | 35,074 | 1,721,408 | 22,465 | 1,102,567 |
| 5 | 2 local and 3 systemic | 49.1 | 66,098 | 3,244,045 | 11,381 | 558,572 |
|  | 3 local and 2 systemic | 39.0 | 56,461 | 2,204,712 | 5,502 | 214,844 |
| 6 | 3 local and 3 systemic | 49.1 | 42,767 | 2,098,976 | 1,869 | 91,729 |
| **Total:** | | **586** | **451,746** | **17,052,869** | **417,129.00** | **11,769,430** |

USD, $1 equal to S/ 3.374 Peruvian Soles (average accounting exchange rate reported on December 31, 2018 by the Peruvian Superintendence of Banks, Insurance and Private Pension Fund Administrators [Superintendencia de Banca, Seguros y AFP]).^20^

AR, adverse reaction; USD, US dollar.

Supplementary Table 10. Estimated total annual costs (USD, $) of detecting and treating OPV-derived polio

| **Variant** | **Variant cost (USD, $)** | **Number of cases** | **Total cost  (USD, $)** | |
| --- | --- | --- | --- | --- |
| Identification and notification of suspected AFP cases | 271,839 | Fixed cost | 271,839 | |
| Diagnosis and monitoring of suspected AFP cases | 4,762 | 55 | 261,905 | |
| Confirmation of probable polio cases | 322 | 1* | 322 | |
| Confirmed polio case – Paraplegia with preserved sensitivity | 4,574 | 1 | 4,574 | |
| Confirmed polio case – Postural disorders or postural scoliosis | 1,597 | 1 | 1,597 | |
| **Total:** | | | | **540,238** |

USD, $1 equal to S/ 3.374 Peruvian Soles (average accounting exchange rate reported on December 31 2018 by the Peruvian Superintendence of Banks, Insurance and Private Pension Fund Administrators [Superintendencia de Banca, Seguros y AFP]).^20^

*Samples for the five probable cases are assumed to be sent to the foreign laboratory together.

AFP, acute flaccid paralysis; OPV, oral polio vaccine; USD, US dollar.

Supplementary figures

Supplementary figure 1. Distribution of infants who present adverse reactions to the vaccines according to the vaccination scheme
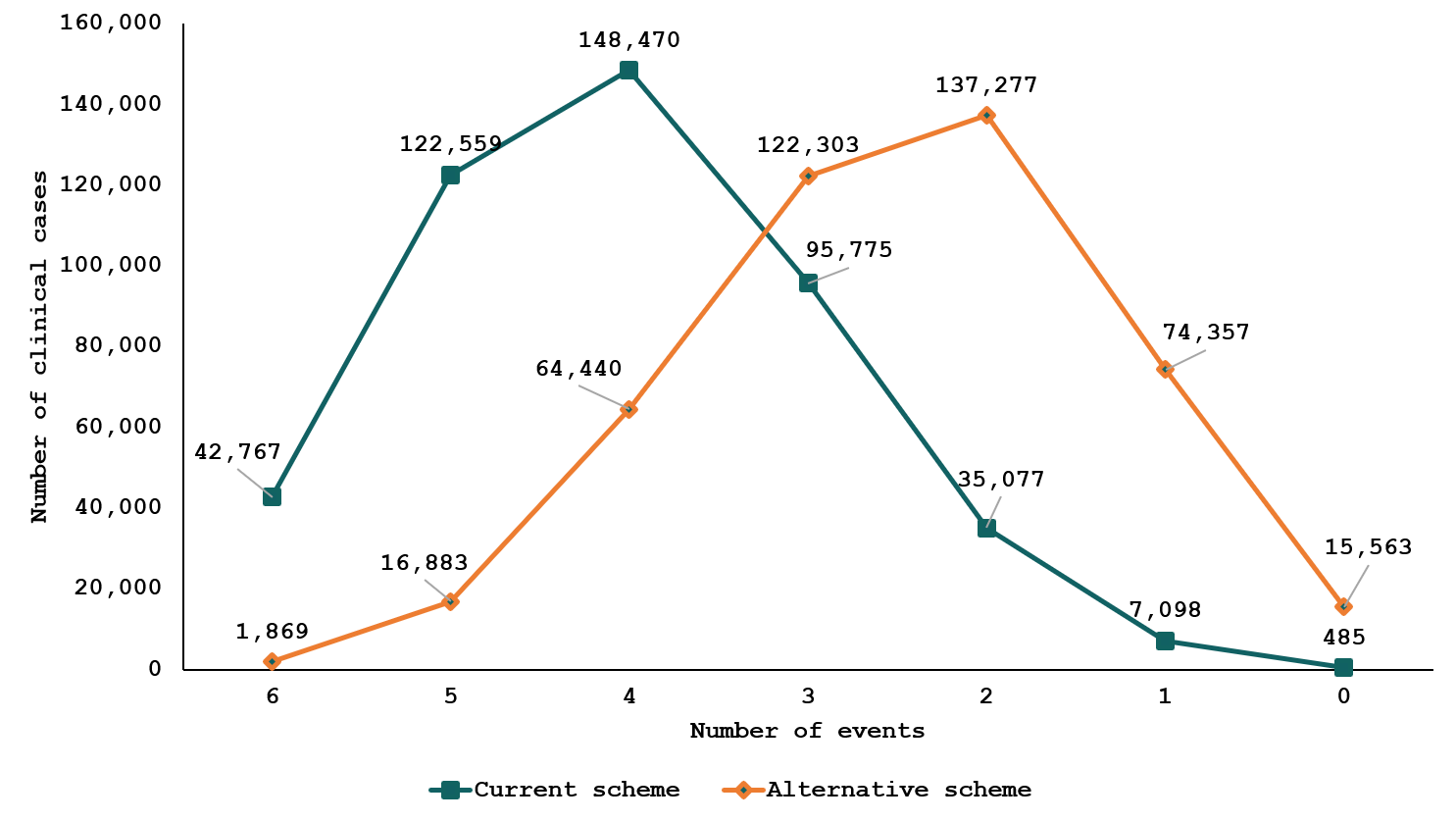


References

1. Gobierno Regional de Lima. Personal Setiembre. 2017 [cited Nov 12, 2020]. Available from: <https://www.diresalima.gob.pe/diresa-antiguo/descargar/transparencia/2017/personal/Personal_Setiembre_2017.pdf>.

2. Ministerio de Economía y Finanzas. Manual de Usuario - Módulo de Logística. [cited Nov 12, 2020]. Available from: <https://www.mef.gob.pe/es/siga/manuales?id=3630>.

3. Ministerio de Salud (MINSA) Republica del Peru. Informe segun directiva n° 010 - minsa/ogc-v.1 proceso de seleccion marzo. 2008 [cited Nov 12, 2020]. Available from: <http://webcache.googleusercontent.com/search?q=cache:lD2EX_jvL74J:ftp://ftp2.minsa.gob.pe/descargas/transparencia/03AdquisicionBS/Archivos/Bieness/05DisaEste/2008/05DisaEste-PC-0308.xls+&cd=1&hl=en&ct=clnk&gl=pe>.

4. Ministerio de Salud (MINSA) Republica del Peru. Resolución Ministerial N° 1231-2018-MINSA. 2018 [cited Nov 12, 2020]. Available from: <https://cdn.www.gob.pe/uploads/document/file/300159/d233380_opt.PDF>.

5. Ministerio de Salud (MINSA) Republica del Peru. Adquisiciones y contrataciones: Gastos de telefonía. 2020 [cited Nov 12, 2020]. Available from: <https://www.minsa.gob.pe/transparencia/index.asp?op=913>.

6. Instituto Nacional de Estadística e Informática (INEI). Perú resultados difinitivos de los censos nacionales 2017: Tomo I. Lima, Peru: Instituto Nacional de Estadística e Informática (INEI); 2018.

7. Gerencia de Salud La Libertad. Población asignada, por edades y grupos especiales, según provincias, distritos y establecimientos de salud - la libertad - 2018 [Internet]. 2018 [cited 2020 Jan 20]. Available from: <http://app.diresalalibertad.gob.pe/HIS/2015/Poblaciones/POBLACION_2018.xlsx>.

8. Gobierno Regional de Arequipa. Población 2018 distrito establecimiento [Internet]. 2018 [cited 2020 Jan 20]. Available from: <http://www.saludarequipa.gob.pe/oei/archivos/2.%20Poblacion/2018/POBLACION%20%202018%20DISTRITOS%20%20ESTABLECIMIENTOS.xls>.

9. Ministerio de Salud (MINSA) Republica del Peru. Padron nominal poblacion de niños menores 6 años [Internet]. 2019 [cited 2020 Jan 20]. Available from: <ftp://ftp.minsa.gob.pe/OEI/Poblacion/Poblacion%20menor%20de%2006%20a%F1os%2031_12_18/PADRON%20NOMINAL%20Poblacion%20de%20ni%F1os%20menores%206%20a%F1os.xlsx>.

10. Ministerio de Salud (MINSA) Republica del Peru. Resolución ministerial N° 195-2009-MINSA; documento técnico “metodología para la estimación de costos estándar en los establecimientos de salud”. 2009 [cited 2020 Jan 20]. Available from: <https://cdn.www.gob.pe/uploads/document/file/275608/246869_RM195-2009EP.pdf20190110-18386-eaam0o.pdf>.

11. Instituto Nacional de Estadística e Informática. Peru encuesta demográfica y de salud familiar-ENDES 2017 [Internet]. 2018 [cited 2020 Jan 20]. Available from: <https://www.inei.gob.pe/media/MenuRecursivo/publicaciones_digitales/Est/Lib1525/index.html>.

12. Patterson J, Kagina BM, Gold M, Hussey GD, Muloiwa R. Comparison of adverse events following immunisation with acellular and whole-cell pertussis vaccines: a systematic review. Vaccine. 2018;36(40):6007–16.

13. Ministerio de Salud (MINSA) Republica del Peru. Directiva Sanitaria No 059-MINSA/DGE-V-01: Directiva Sanitaria Para La Vigilancia Epidemiológica De Poliomielitis /Parálisis Flácida Aguda. 2015 [cited 2020 Jan 17]. Available from: <https://cdn.www.gob.pe/uploads/document/file/373711/Directiva_sanitaria_para_la_vigilancia_epidemiol%C3%B3gica_de_poliomielitispar%C3%A1lisis_fl%C3%A1cida_aguda20190925-31433-3e9hij.pdf>.

14. Vargas Huapaya MA. Dirección de Salud II Lima Sur Oficina de Epidemiología: Vigilancia epidemiológica. 2014.

15. Ministerio de Economía y Finanzas. Anexo N° 03: Parámetros de Evaluación Social de la Directiva N° 002-2017-EF/63.01. 2017 Available from: <https://www.mef.gob.pe/es/anexos-y-formatos>.

16. Instituto Nacional de Estadística e Informática (INEI), Presidencia del Consejo de Ministros (PCM), Dirección Técnica de Demografía e Indicadores Sociales (DTDIS) - INEI. Perú - Encuesta nacional de hogares sobre condiciones de vida y pobreza 2017. 2017 [cited 2020 Jan 16]. Available from: <https://www.ilo.org/surveydata/index.php/ddibrowser/2027/export/?format=pdf&generate=yes>.

17. Ministerio de Salud (MINSA) Republica del Peru. NTS Nº 141 - MINSA/2018/DGIESP norma técnica de salud que establece el esquema nacional de vacunaciόn (Resoluciόn Ministerial No 719-2018/MINSA). Lima, Peru: Ministerio de Salud (MINSA) Republica del Peru; 2018.

18. Sanofi Pasteur. Hexaxim Summary of Product Characteristics. 2017.

19. Santos-Lima E, B'Chir S, Lane A. Combined immunogenicity data for a new DTaP-IPV-Hep B-PRP-T vaccine (Hexaxim) following primary series administration at 2, 4, 6 months of age in Latin America. Vaccine. 2013;31(9):1255–8.

20. United States Security and Exchange Commission. Annual report pursuant to section 13 or 15(d) of the securities exchange act of 1934 for the fiscal year ended December 31, 2018. Washington, DC: 2018 [cited 2020 Jan 20]. Available from: <https://www.sec.gov/Archives/edgar/data/1221029/000161577419006749/s117247_20f.htm>.
